# Conformational and Physicochemical Properties of *Lagenaria siceraria* Seed Protein Isolate‐Cocoyam Mucilage Conjugates: Impact of Glycation Time

**DOI:** 10.1002/fsn3.71933

**Published:** 2026-05-23

**Authors:** Gbenga Isaac Oluwafemi, Eunice Moriyike Ogunbusola, Kunle Oni, Opeyemi Olaitan Alabi, Kudirat Titilope Araoye

**Affiliations:** ^1^ Department of Food Science and Technology Federal Polytechnic Ado Ekiti Ado Ekiti Nigeria; ^2^ Department of Food Science and Technology Federal University Oye‐Ekiti Ekiti Nigeria; ^3^ Department of Biotechnology and Food Science Durban University of Technology Durban South Africa

**Keywords:** cocoyam‐mucilage, DSC, FTIR, *Lagenaria siceraria* protein, Maillard reaction

## Abstract

The growing popularity of plant proteins as food ingredients can be attributed to their high nutritional content and sustainability. However, their usage is restricted because of their limited functionality. This study investigated the structural conformation and functional properties of *Lagenaria siceraria* protein isolate‐cocoyam mucilage conjugates glycated at 24, 48, and 72 h, respectively. The conjugates were analyzed for proximate composition, amino acid and structural conformation, physicochemical, and some functional properties employing standard methods. The results showed that *Lagenaria siceraria* protein conjugates had improved ash and crude fiber content. Notably, the conjugation with cocoyam mucilage significantly increased the levels of arginine and valine. Increased absorbance observed at 304 nm confirmed the formation of early intermediate Amadori compounds in the conjugates. The α‐helix structure increased while the β‐sheet decreased as the glycation time advanced. The conjugates showed higher denaturation temperature compared to the protein isolate (84°C), indicating an improved resistance to thermal stress. Prolonged conjugation period between *Lagenaria siceraria* protein isolate and cocoyam mucilage enhanced the morphology of the modified protein. The solubility profile of the conjugates increased significantly compared to the protein isolate at the pH ranges under investigation. The emulsion activity of the conjugates was considerably increased, while the emulsion was significantly stabilized at every pH that was tested; the foaming qualities were noticeably enhanced. In this research, conjugating *Lagenaria siceraria* proteins with cocoyam mucilage has advantageous effects that improve solubility at higher pH levels. Overall, the results show that these conjugated proteins could be used as novel ingredients in food applications.

## Graphical abstract

Maillard conjugates showed a higher denaturation temperature compared to the protein isolate. It had a higher solubility profile compared to the protein isolate. It increased the emulsifying and foaming properties of *Lagenaria siceraria* protein isolate.

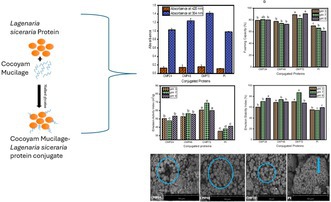

## 1 Introduction

Plant proteins are in high demand globally because of the higher cost of animal proteins, their low‐density lipoprotein content, and sustainability (Barnard and Leroy 2020). Soybeans and peas have been widely used in the food industry as emulsifiers, foaming agents, and thickeners (Deng 2021; Goldstein and Reifen 2022; Shanthakumar et al. 2022). However, climate change is adversely affecting the annual production of these crops, including soybeans and peas, which are widely used as ingredients in the food system (Siamabele 2021). There are also many underutilized drought‐tolerant legumes, such as bottle gourd melon seed [ *Lagenaria siceraria* (LS)].

The poor functional properties of plant proteins, as well as their stability in their native state, limit their use as food ingredients in the food system (Tan et al. 2023). In response to the limitations associated with the use of plant proteins as techno‐functional ingredients, several modification methods, including physical, chemical, and enzymatic approaches, have been used to improve the functional properties of plant proteins and expand their industrial food applications (Akharume et al. 2021; Glusac and Fishman 2021; Ogunbusola et al. 2023; Rashwan et al. 2025; Tan et al. 2023). Previous studies have reported residual effects of chemicals/reagents employed in modifying plant proteins, which are detrimental to health (Kutzli et al. 2021). Plant proteins have been hydrolyzed with various enzymes, such as trypsin and pepsin (Tawalbeh et al. 2023). The hydrolysis process alters the structure of the protein but can impart a bitter taste (Cui et al. 2021), thereby limiting the application of enzymatically hydrolyzed protein in the food system (Akharume et al. 2021; Zhang et al. 2018). One of the most promising methods to achieve techno‐functional improvement of plant protein without impacting bitter taste or leaving chemical residues is glycation under the influence of heat via the Maillard reaction. It requires no use of chemicals (Monika and Sarika 2020).

Glycation, a spontaneous non‐enzymatic reaction, takes place between an available amino group and a carbonyl‐containing moiety, usually a reducing sugar (Kutzli et al. 2021). Glycation with glucose, a carbohydrate, increased the solubility of proteins from bitter melon seeds by 90% and improved their emulsifying and foaming qualities (Horax et al. 2014). Furthermore, after oil extraction, the defatted walnut flour was used to make walnut proteins, which were then glycated with glucose to improve the conjugated samples' emulsifying activity and stability (Ullah et al. 2019). This was done without characterizing the time‐dependent name of the protein‐mucilage conjugates.

The objective of this study is to investigate the structural conformation, thermal characteristics, and functional characteristics (solubility, emulsifying, and foaming qualities) of conjugates of *Lagenaria siceraria* protein isolate and cocoyam mucilage glycated at 24, 48, and 72 h, respectively.

## 2 Materials and Procedures

### 2.1 Materials

The seeds for the bottle gourd melon ( *Lagenaria siceraria* ) were purchased from Saki Sango market in Oyo State, Nigeria. The cocoyam was bought at Ado Ekiti's Oja Bisi market. All chemicals used for this experiment were of food grade and supplied by Bisolab Ajilosun Ado Ekiti, Nigeria.

### 2.2 Procedures

#### 2.2.1 The Process of Making Defatted Flour

Dehulled *Lagenaria siceraria* seeds were ground into flour and then defatted with *n*‐hexane in a 1:10 (g/ml) (flour: solvent) ratio for 3 h, as explained by Arise et al. (2014). To get rid of any remaining hexane, the defatted flour was kept in a fume cupboard overnight before being stored at 4°C.

#### 2.2.2 Protein Isolates' Preparation

Protein isolates from *Lagenaria siceraria* were made using the procedures outlined by Li et al. (2021) and Ogunbusola et al. (2013). Distilled water was combined with defatted seed flour at a 1:20 w/v meal‐to‐solvent ratio. After ten minutes of stirring with a magnetic stirrer, 0.1 M HCl or 0.1 M NaOH was added dropwise to bring the slurry's pH down to 9.0. To guarantee complete mixing, the extraction process was stirred continuously for 2 h. To separate the leftovers, the slurry was then centrifuged for 20 min at 4000 × *g*. After collecting the supernatants, proteins were precipitated by using 0.1 M HCl to bring the pH down to 4.5. For an additional twenty minutes, the sample was centrifuged at 4000 × *g*. The resulting protein residue was then dialyzed against distilled water using cellulose membrane dialysis tubing (D9402‐100FT, Sigma‐Aldrich USA) with an average flat width of 76 mm. This process was carried out for eighteen hours at room temperature. Throughout the procedure, the water was replaced every four hours. A freeze drier was used to collect and freeze‐dry the precipitate (MODEL NO. LGL‐10, Searhech Instruments). The dried protein was then stored at 4°C until further use.

#### 2.2.3 Mucilage Extraction

Fresh *Colocasia esculenta* rhizomes were gathered, rinsed with portable water, peeled, and cleaned with water. To fully release the mucilage into the water, the tubers were crushed, steeped in water for six hours, boiled for thirty minutes, and then allowed to stand for one hour. The marc was taken out of the solution by extracting the mucilage with a muslin cotton bag. To precipitate the mucilage, acetone was added in proportions three times the filtrate volume. According to Tosif et al. (2023), the mucilage was separated, dried in an oven at 40°C, gathered, crushed, and then put through an 80 μm filter before being kept in a desiccator at 30°C and 45% relative humidity until it was needed.

#### 2.2.4 Conjugates of the *Lagenaria siceraria* Protein Isolate and Cocoyam Mucilage

According to Li et al. (2020), *Lagenaria siceraria* protein isolate and cocoyam mucilage powder were mixed in distilled water at a weight ratio of 1:2 and agitated for four hours at 25°C. To create the mixture, this solution was lyophilized and then carefully ground into a fine powder. The mixed samples were put in a desiccator and incubated at 60°C for 24, 48, and 72 h, respectively, to produce conjugates of the *Lagenaria siceraria* protein isolate and cocoyam mucilage. A saturated KBr solution kept the relative humidity at 79%. Samples were extracted at 24, 48, and 72 h, in that order. Before being analyzed further, all conjugates were freeze‐dried and kept at 4°C.

### 2.3 Proximate Composition

The proximate analysis of the sample's moisture, ash, fiber, fat, protein, and the carbohydrate was determined by difference: 100 − (Moisture + Ash + Fat + Fiber) using the standard methods of AOAC (2012).

### 2.4 Assessment of the Content of Amino Acids

The Applied Biosystems PTH Amino Acid Analyzer was used to determine the amino acid profiles of the *Lagenaria siceraria* protein isolate and its conjugates (Bidlingmeyer et al. 1984). 6 mol/L HCl was used to hydrolyze the materials for 24 h at 116°C. Tryptophan content was measured following alkaline hydrolysis (Landry and Delhaye 1992), whilst cysteine and methionine concentrations were assessed via performic acid oxidation (Gehrke et al. 1985). The digests were separated using a gradient of sodium citrate buffers (pH 3.45 and pH 10.85) at a flow rate of 0.45 mL per minute on an Agilent ZORBAX Eclipse XDB C18 column, USA (4.6 × 150 mm).

### 2.5 The Browning Index

As explained by Shen and Li (2021), the *Lagenaria siceraria* protein isolate and its conjugates were used to measure the browning process. Protein isolate‐cocoyam mucilage conjugates' amadori compound and melanoidin production are thought to be indicated by UV absorbances at 304 and 420 nm, respectively. One (1) mg/mL of A solution of 0.1% (w/v) sodium dodecyl sulfate was used to dilute the samples five times. After which, the supernatant was gathered and examined using a double beam spectrophotometer (VWR UV‐6300PC, VWR International, Radnor, PA, USA) at 304 and 420 nm.

### 2.6 Differential Scanning Calorimetry (DSC)

According to a modified version of Alabi et al. (2020), the thermal properties of *Lagenaria siceraria* protein isolate and conjugates were investigated using a differential scanning calorimeter (model DSC‐7, PerkinElmer, Norwalk, CT, USA). 2.5 mL of 0.05 mol/L sodium phosphate buffer (pH 7) was combined with 1 g of the sample. Twenty milligrams of the mixture were carefully weighed and put into each aluminum pan after it had been let to sit at 4°C for 12 h. The pans were hermetically sealed and heated from 20to 200°C at a rate of 5°C/min, while an empty, sealed pan served as a reference.

### 2.7 Fourier Transformed Infrared (FT‐IR)

In accordance with the methods of Ruzengwe et al. (2020) and Alabi et al. (2023), the FT‐IR spectra of the conjugates and protein isolate were evaluated. Using potassium bromide (KBr) salt, the samples were ground into a fine powder before being compressed under vacuum. At a resolution of 4 cm, the samples were scanned between 4000 and 400 cm^−1^. Using the fourier self‐deconvolution technique, the data in the amide I region (1700–1600 cm–1) was deconvoluted to yield the secondary structure. To more precisely identify the various secondary structure elements found in the protein, a mathematical procedure was implemented to separate the overlapping components of the amide I band.

### 2.8 Scanning Electron Microscopy

Following a previously published procedure by Klost and Drusch (2019), the surface morphology of *Lagenaria siceraria* protein isolate and *Lagenaria siceraria* protein isolate‐cocoyam mucilage gels were prepared at a 22.5% concentration and analyzed using scanning electron microscopy (SEM, Zeiss Ultra Plus FEG SEM, Zeiss, Germany) at an accelerating voltage of 5 kV.

### 2.9 Functional Properties

#### 2.9.1 Determination of Emulsion Capacity and Stability

The emulsion capacity and stability were assessed using the approach outlined by Pirestani et al. (2017). A protein solution containing 2 mg/mL was made using 10 mmol/L of phosphate buffer (pH 7.0). The protein samples were then mixed with soybean oil at a 1:3 (v/v) ratio. A homogenizer (AE300L‐H, Shanghai Angni Instruments Co.) was used to homogenize the mixtures for 10 min at 24,000 rpm. 50 μL of the resulting emulsion was pipetted at a 1:100 ratio into a sodium dodecanesulfonate solution (1 mg/mL). A wavelength of 500 nm was used to measure the diluted emulsion's absorbance. The following formula was used to determine the emulsification activity index (EAI) and emulsification stability index (ESI) values:

$$\mathrm{EAI}=\frac{2\times 2.303\times A0\times \mathrm{Df}}{C\times \phi \times \theta \times 1000}$$

$$\mathrm{ESI}=\frac{Åo\times 10}{A_{0}-A_{10}}$$

*A*_0_ and *A*_10_ were the absorbance readings of the emulsion at 0 and 10 min, respectively, where DF is the dilution factor (100), C is the protein concentration (g/mL), φ is the optical path (1 cm), and θ is the oil volume fraction (0.25).

#### 2.9.2 Assessment of the Solubility of Proteins

Using the method developed by Ogunbusola et al. (2023), the protein solubility was assessed. Distilled water was used to disperse the samples until the final protein concentration was 2 mg/mL. The protein solutions were then treated with either 0.5 M HCl or 0.5 M NaOH to raise their pH from 3 to 9. Following these modifications, the solutions underwent a 30 min, 12,000‐*g* centrifugation at 4°C. Lowry's method was used to calculate the final supernatant's protein content.

#### 2.9.3 The Foaming Capacity and Stability

Protein dispersions at different concentrations of 10, 15, and 20 mg/mL (protein weight basis) were prepared in 0.1 M phosphate buffer, pH 7, in order to measure foam capacity. The polytron PT 3100 homogenizer (Kinematica AG, Lucerne, Switzerland) was used to homogenize these at 20,000 rpm for one minute with a 20 mm foaming shaft (Adebiyi and Aluko 2011). Hence, the foaming capacity was determined:

$$\mathrm{FC}\%=\frac{\text{Volume after homogenization}-\text{Volume before homogenization}}{\text{Volume before homogenization}}$$

### 2.10 Statistical Analysis

The statistical program SPSS version 23 was used to analyze the data, which was presented as the mean ± standard deviation of three measurements. Duncan's test was used to determine a significant difference at *p* < 0.05 after a one‐way analysis of variance (ANOVA) was performed.

## 3 Discussion

### 3.1 Proximate Composition of Protein Isolate and Its Conjugates From *Lagenaria siceraria*

The nutritional profiles of the samples are provided by their proximate composition, as shown in Table 1, which also shows how glycation time affects the *Lagenaria siceraria* protein isolate (PI) and its conjugates with cocoyam mucilage. Protein content of PI is noticeably higher than that of the conjugated samples (CMP24, CMP48, and CMP72). This is probably because the conjugation with cocoyam mucilage introduces more carbohydrates and crude fiber, which dilutes the protein concentration. Moreso, longer conjugation periods may slightly reduce protein content due to Maillard reactions under prolonged glycation conditions, as demonstrated by the conjugated samples' decreased protein content. The variations in the proximate composition can be attributed to excessive Maillard progression, aggregation effects, and structural collapse at longer reaction times. *Lagenaria siceraria* protein isolates could be a potential protein supplement in food formulation compared to β‐glucan conjugates between oat protein isolate and *Pleurotus ostreatus* (Li et al. 2021). It could also be an alternative replacement for animal proteins. Protein content decreased as the glycation time increased. It was recorded that glycation via Maillard reaction increased the ash, moisture, crude fiber, and fat content of the protein isolate. The data obtained in this study indicated that the *Lagenaria siceraria* protein isolate‐cocoyam mucilage conjugates might have increased bulk density, which would be more appropriate for low‐calorie, high‐fiber products as well as culinary applications where texture and volume are desired.

**TABLE 1:** Proximate composition (%) of *Lagenaria siceraria* protein isolate‐cocoyam mucilage conjugates.

Sample	Crude protein	Moisture	Fat	Total ash	Crude fiber	Carbohydrate
CMP24	53.47^b^ ± 0.32	6.65^b^ ± 0.22	1.83^a^ ± 0.58	2.69^a^ ± 0.18	1.19^b^ ± 0.02	34.17^a^ ± 0.13
CMP48	49.76^d^ ± 0.14	7.25^a^ ± 0.14	1.08^c^ ± 0.85	2.84^a^ ± 0.11	1.23^b^ ± 0.11	37.85^a^ ± 0.85
CMP72	51.81^c^ ± 0.44	6.74^b^ ± 0.26	1.70^b^ ± 0.15	2.46^c^ ± 0.23	1.81^a^ ± 0.10	35.48^a^ ± 0.31
PI	91.22^a^ ± 1.02	2.06^c^ ± 1.43	0.67^c^ ± 0.01	1.84^b^ ± 1.33	1.20^b^ ± 1.35	1.34^b^ ± 0.35

*Note:* *N* = 3; values are mean ± SD. There was a significant difference (*p* < 0.05) between values with different superscripts on the same column. CMP24: *Lagenaria siceraria* seed protein isolate‐cocoyam mucilage conjugate at 24 h. CMP48: *Lagenaria siceraria* seed protein isolate‐cocoyam mucilage conjugate at 48 h. CMP72: *Lagenaria siceraria* seed protein isolate‐cocoyam mucilage conjugate at 72 h. PI = *Lagenaria siceraria* seed protein isolate.

### 3.2 Amino Acid Composition

Glutamic acid, aspartic acid, and lysine were the major amino acids present in the *Lagenaria siceraria* protein isolate, similar to the *Lagenaria siceraria* protein isolate‐cocoyam mucilage Maillard conjugate (Table 2). The lysine content of the protein isolate decreased significantly by about 40% after glycation via the Maillard reaction over the studied duration. This is evidence of a condensation reaction between the carbonyl groups of the reducing sugar from cocoyam mucilage and the ε‐amino groups of lysine (Kutzli et al. 2021). It also indicates that lysine provides the major binding site (Zhong et al. 2019) for the Maillard reaction. The preference for the lysine residue over arginine as the active binding site in the *Lagenaria siceraria* protein isolate might be attributed to the structural composition and conformation of the cocoyam mucilage (Khan et al. 2023). A significant reduction in the amount of tryptophan was recorded, similar to the histidine content (Rudolph et al. 2018). This might be related to interactions between the α‐amino groups of terminal amino acids and the indole and imidazole groups of tryptophan and histidine, respectively (Rudolph et al. 2018). In addition, the reduction in amino acid content might be attributed to thermal degradation (Xiao et al. 2021) during the glycation process. Arginine was recorded to increase as the glycation time increased, contrary to the report of Tao et al. (2024). Tao et al. (2024) reported that most hydrophilic amino acids were involved in the Maillard reaction. The value recorded for methionine (a limiting amino acid in most legumes) in *Lagenaria siceraria* and its Maillard conjugates was higher than that reported for other grains. The values recorded for both the essential and non‐essential amino acids were higher than the recommended values by FAO/WHO (2007). The variations in the amino acids composition can be attributed to excessive Maillard progression, aggregation effects, and structural collapse at longer reaction times.

**TABLE 2:** Amino acid profile of *Lagenaria siceraria* seed protein isolate and its conjugates (g/100 g protein).

Sample	PI	CMP24	CMP48	CMP72	FAO/WHO (2007)
Leucine	10.46^a^ ± 0.09	7.67^b^ ± 0.02	7.17^bc^ ± 0.04	6.75^c^ ± 0.04	6.10
Lysine	7.54^a^ ± 0.06	4.71^b^ ± 0.60	4.69^b^ ± 0.46	4.11^b^ ± 0.61	4.80
Isoleucine	6.24^a^ ± 0.03	4.08^c^ ± 0.05	4.18^b^ ± 0.02	3.39^d^ ± 0.02	3.00
Phenylalanine	4.00^c^ ± 0.01	4.47^a^ ± 0.02	4.14^b^ ± 0.06	3.62^d^ ± 0.02	1.90
Trytophan	2.29^a^ ± 0.02	0.73^c^ ± 0.04	0.82^b^ ± 0.02	0.63^d^ ± 0.02	0.66
Valine	2.29^d^ ± 0.02	4.59^a^ ± 0.02	4.34^b^ ± 0.02	3.95^c^ ± 0.04	4.00
Methionine	3.19^a^ ± 0.02	1.37^c^ ± 0.02	1.42^b^ ± 0.04	1.22^d^ ± 0.02	1.30
Proline	4.57^a^ ± 0.04	3.24^b^ ± 0.01	3.08^c^ ± 0.02	3.12^c^ ± 0.01	0.55
Arginine	3.62^d^ ± 0.02	6.33^a^ ± 0.04	5.78^b^ ± 0.11	5.14^c^ ± 0.07	—
Tyrosine	3.19^a^ ± 0.02	3.18^a^ ± 0.02	2.93^b^ ± 0.03	2.44^c^ ± 0.05	0.33
Histidine	2.91^a^ ± 0.02	2.68^b^ ± 0.02	2.34^d^ ± 0.03	2.52^c^ ± 0.03	1.90
Cystine	2.25^a^ ± 0.02	1.14^b^ ± 0.02	1.02^c^ ± 0.02	0.79^d^ ± 0.08	—
Alanine	5.58^a^ ± 0.03	4.03^c^ ± 0.02	4.28^b^ ± 0.03	3.57^c^ ± 0.05	—
Glutamic Acid	16.64^a^ ± 0.06	13.40^b^ ± 0.04	13.03^c^ ± 0.03	12.21^d^ ± 0.09	9.90
Glycine	2.86^b^ ± 0.04	3.12^a^ ± 0.05	2.64^c^ ± 0.03	2.84^b^ ± 0.02	0.55
Threonine	4.18^a^ ± 0.02	3.16^b^ ± 0.03	3.05^c^ ± 0.02	2.69^d^ ± 0.05	2.50
Serine	5.95^a^ ± 0.23	4.11^b^ ± 0.04	4.03^b^ ± 0.02	3.54^b^ ± 0.05	0.55
Aspartic Acid	9.65^a^ ± 0.03	9.03^c^ ± 0.02	9.24^b^ ± 0.02	8.78^d^ ± 0.04	0.65

*Note:* *N* = 3; values are mean ± SD. There was a significant difference (*p* < 0.05) between values with different superscripts in the same column. CMP24: *Lagenaria siceraria* seed protein isolate‐cocoyam mucilage conjugate at 24 h. CMP48: *Lagenaria siceraria* seed protein isolate‐cocoyam mucilage conjugate at 48 h. CMP72: *Lagenaria siceraria* seed protein isolate‐cocoyam mucilage conjugate at 72 h. PI = *Lagenaria siceraria* seed protein isolate.

### 3.3 Quantification of Amadori Compounds and Browning

**FIGURE 1:**
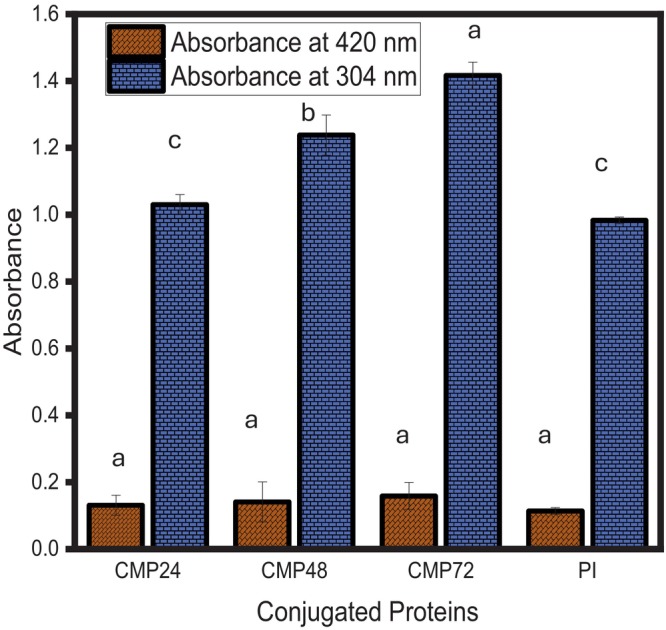
Browning index of *Lagenaria siceraria* seed protein isolate‐cocoyam mucilage conjugate*s*. *N* = 3; values are mean ± SD. Bars with different alphabets are statistically different (*p* < 0.05) at the studied absorbance. CMP24: *Lagenaria siceraria* seed protein isolate‐cocoyam mucilage conjugate at 24 h. CMP48: *Lagenaria siceraria* seed protein isolate‐cocoyam mucilage conjugate at 48 h. CMP72: *Lagenaria siceraria* seed protein isolate‐cocoyam mucilage conjugate at 72 h. PI = *Lagenaria siceraria* seed protein isolate.

There was a significant increase in the development of Amadori compounds (absorbance measured at 304 nm) in the *Lagenaria siceraria* protein isolate‐cocoyam mucilage Maillard conjugate compared to the protein isolate (Figure 1). The formation of Amadori compounds progressed as the Maillard reaction time increased from 24 to 72 h. A lower development of browning (absorbance measured at 420 nm) was observed for all the Maillard conjugates, even at higher exposure time (72 h), compared to the protein isolate. The formation of Amadori compounds and the development of browning suggest that Maillard reaction occurred in the glycated samples (Keel et al. 2022). The formation of Amadori compounds and browning, indicating progression of the Maillard reaction, were consistent with the decrease in the amount of lysine in the conjugates, as shown in Table 2. This suggested the formation of *Lagenaria siceraria* protein isolate‐cocoyam mucilage Maillard conjugates. The higher browning compared to the protein isolate suggested more advanced glycation end products or melanoidins. The data obtained in this study followed a similar trend to that reported for *Cinnamomum camphora* protein isolate‐dextran Maillard conjugate (Yan et al. 2022).

### 3.4 The Secondary Structure of the Protein Isolates and Its Conjugates From *Lagenaria siceraria* Seeds

The dominant secondary structure in the *Lagenaria siceraria* protein isolate‐cocoyam mucilage Maillard conjugates is the β‐sheet structure similar to that of the protein isolate (Table 3). The α‐helix content of the *Lagenaria siceraria* protein isolate‐cocoyam mucilage Maillard conjugates was higher than that of the protein isolate. The β‐sheet content decreased while the α‐helix content increased as conjugation time increased from 24 to 72 h. This pattern may reflect the increase in the ordered structure of the protein, which is consistent with the increased ΔH value observed with DSC in Table 4 (Yan et al. 2022). The secondary structure values after deconvolution may be attributed to polysaccharides bound to the ε‐amino group in lysine (Yan et al. 2022). It may also suggest glycoprotein aggregation and changes in the spatial conformation of the protein molecule (Wen et al. 2020). A significant increase was observed in the random coil content of the conjugates. The secondary structure data are similar to those reported by Song et al. (2022). The data may indicate enhanced surface hydrophilicity of the conjugated samples (Li et al. 2020).

**TABLE 3:** Secondary structure (%) of conjugated *Lagenaria siceraria* protein isolate‐cocoyam mucilage Maillard conjugates.

Sample	β‐Sheet (%)	α‐Helix (%)	β‐Turns (%)	Random coils (%)
CMP24	56.78^b^ ± 0.12	21.37^c^ ± 0.19	18.96^a^ ± 0.24	2.89^c^ ± 0.45
CMP48	51.62^c^ ± 0.89	25.78^b^ ± 0.09	12.12^c^ ± 0.12	10.48^b^ ± 0.34
CMP72	50.56^c^ ± 0.06	27.56^a^ ± 0.12	8.98^d^ ± 0.17	12.90^a^ ± 0.10
PI	62.23^a^ ± 0.09	21.11^c^ ± 0.78	15.78^b^ ± 0.05	0.88^d^ ± 0.19

*Note:* Values are mean ± SD. *N* = 3; values are mean ± SD. There was a significant difference (*p* < 0.05) between values with different superscripts in the same column. CMP24: *Lagenaria siceraria* seed protein isolate‐cocoyam mucilage conjugate at 24 h. CMP48: *Lagenaria siceraria* seed protein isolate‐cocoyam mucilage conjugate at 48 h. CMP72: *Lagenaria siceraria* seed protein isolate‐cocoyam mucilage conjugate at 72 h. PI = *Lagenaria siceraria* seed protein isolate.

**TABLE 4:** Thermal properties of conjugated *Lagenaria siceraria* protein isolate‐cocoyam mucilage Maillard conjugates.

Samples	T_O_ (°C)	Td (°C)	T_C_ (°C)	∆H (J/g)
CMP24	71.16^c^ ± 0.12	99.21^b^ ± 0.00	123.21^c^ ± 0.08	10.54^c^ ± 0.09
CMP48	79.45^b^ ± 0.01	110.10^a^ ± 0.03	132.05^b^ ± 0.06	12.57^b^ ± 0.05
CMP72	85.54^a^ ± 0.14	113.04^a^ ± 0.02	141.04^a^ ± 0.09	18.23^a^ ± 0.17
PI	69.43^d^ ± 0.06	84.43^c^ ± 0.06	88.43^d^ ± 0.06	8.43^d^ ± 0.06

*Note:* Values are mean ± SD. *N* = 3; values are mean ± SD. There was a significant difference (*p* < 0.05) between values with different superscripts in the same column. CMP24: *Lagenaria siceraria* seed protein isolate‐cocoyam mucilage conjugate at 24 h. CMP48: *Lagenaria siceraria* seed protein isolate‐cocoyam mucilage conjugate at 48 h. CMP72: *Lagenaria siceraria* seed protein isolate‐cocoyam mucilage conjugate at 72 h. PI = *Lagenaria siceraria* seed protein isolate.

Abbreviations: *T*c, conclusion gelatinization temperatures; *T*o, onset gelatinization temperatures; *T*p, peak gelatinization temperatures; Δ*H*, gelatinization enthalpy.

### 3.5 Thermal Characteristics of Protein Isolates and Conjugates From *Lagenaria siceraria*

**FIGURE 2:**
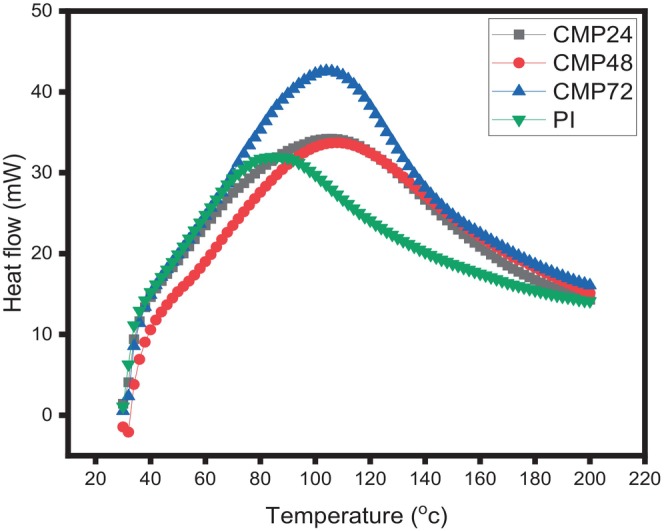
Thermal properties of *Lagenaria siceraria* seed protein isolate‐cocoyam mucilage conjugate*s*. CMP24: *Lagenaria siceraria* seed protein isolate‐cocoyam mucilage conjugate at 24 h. CMP48: *Lagenaria siceraria* seed protein isolate‐cocoyam mucilage conjugate at 48 h. CMP72: *Lagenaria siceraria* seed protein isolate‐cocoyam mucilage conjugate at 72 h. PI = *Lagenaria siceraria* seed protein isolate.

The denaturation temperature of the conjugates increased from 84°C in the protein isolate to 110°C in the conjugates glycated for 24, 48, and 72 h, respectively (Figure 2). This result showed that glycation via maillard reaction significantly improved the thermal stability of the *Lagenaria siceraria* protein isolates‐cocoyam mucilage conjugates. This phenomenon might be linked to the spatial site blocking effect that inhibits the stretching of the protein peptide chains, resulting from the glycation reaction between the sugar chains and the peptide chains on the surface of the protein (Meng et al. 2023). In addition, Cocoyam mucilage as a high molecular weight polysaccharide, thus factors such as steric hindrance, hydration shell effects, and increased viscosity can impact the thermal stabilization of the *Lagenaria siceraria* protein isolates‐cocoyam mucilage conjugates. A higher denaturation temperature (Td) (Table 4) indicated a more condensed tertiary conformation of the polypeptides that suggested an increased resistance to heat‐induced structural changes (Chen et al. 2024). Also, the higher denaturation temperature exhibited by the conjugates indicated a more folded protein structure, which supports the larger α‐helix structure. The data obtained in this study are similar to those reported by Dong et al. (2020). The ΔH values increased as the glycation timing increases, which may reflect the increase in the ordered structure of the protein. as shown in Table 4.

The revised version still attributes thermal stabilization only to glycation without fully considering the effect of the polysaccharide matrix. Since cocoyam mucilage is a high molecular weight polysaccharide, thermal stabilization could also arise from factors such as steric hindrance, hydration shell effects, and increased viscosity. Therefore, expand the discussion to consider physical interactions in addition to covalent glycation.

### 3.6 *Lagenaria siceraria* Protein Isolate and Its Conjugates' Microstructure

**FIGURE 3:**
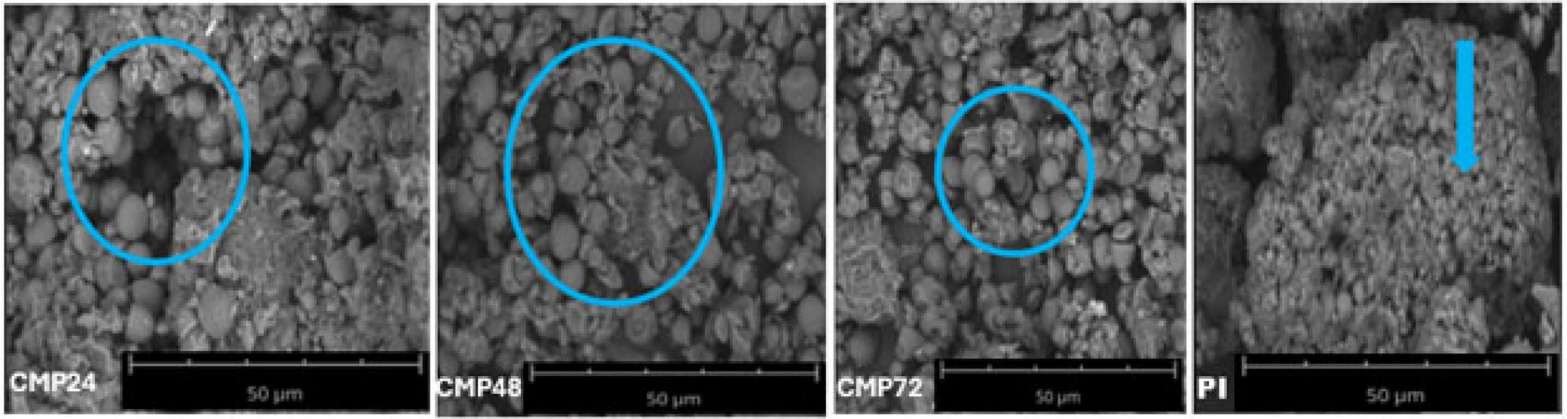
Scanning electron microscopy image of *Lagenaria siceraria* protein isolate‐cocoyam mucilage conjugate. CMP24: *Lagenaria siceraria* seed protein isolate‐cocoyam mucilage conjugate at 24 h. CMP48: *Lagenaria siceraria* seed protein isolate‐cocoyam mucilage conjugate at 48 h. CMP72: *Lagenaria siceraria* seed protein isolate‐cocoyam mucilage conjugate at 72 h. PI = *Lagenaria siceraria* seed protein isolate.

The aggregation state of *Lagenaria siceraria* protein isolate‐cocoyam mucilage Maillard conjugates was observed by SEM (Figure 3). The formation of the Maillard aggregates might be due to the steric block effect of the cocoyam mucilage. The surface of the protein isolate was massive, but the volume of the massive tissue was small. The image showed that the cocoyam mucilage was strongly/covalently linked to the protein isolate, making conjugates. The *Lagenaria siceraria* protein isolate‐cocoyam mucilage Maillard conjugates showed compact and non‐homogeneous microstructure.

### 3.7 Protein Solubility in Conjugated Samples and *Lagenaria siceraria* Protein Isolate

**FIGURE 4:**
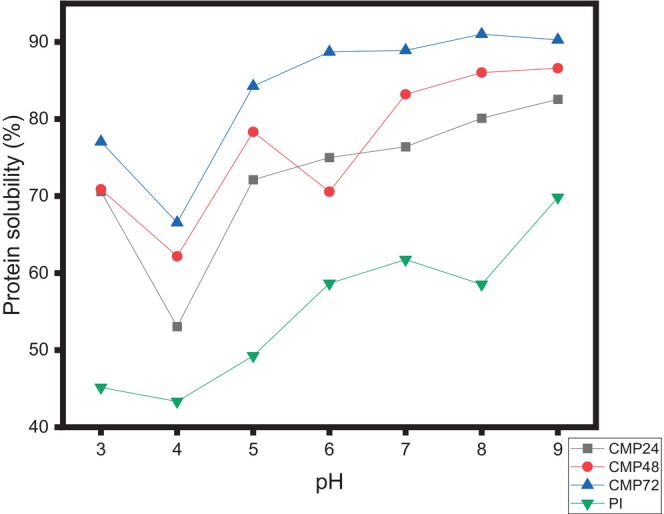
Solubility profile of *Lagenaria siceraria* protein isolate‐cocoyam mucilage conjugate. CMP24: *Lagenaria siceraria* seed protein isolate‐cocoyam mucilage conjugate at 24 h. CMP48: *Lagenaria siceraria* seed protein isolate‐cocoyam mucilage conjugate at 48 h. CMP72: *Lagenaria siceraria* seed protein isolate‐cocoyam mucilage conjugate at 72 h. PI = *Lagenaria siceraria* seed protein isolate.

The *Lagenaria siceraria* protein isolate‐cocoyam mucilage Maillard conjugates showed an improved solubility profile compared to the protein isolate at all studied pHs (Figure 4). The solubility profile of the *Lagenaria siceraria* protein isolate‐cocoyam mucilage Maillard conjugates increased as the glycation time increased from 24 to 72 h. The percentage solubility recorded for *Lagenaria siceraria* protein isolate‐cocoyam mucilage Maillard conjugates glycated at varying times (24, 48, and 72 h) was higher (up to about 68%) at pH 4, a pH close to the isoelectric point of protein isolate. The improved protein solubility profile of the conjugated samples could be attributed to the lower α‐helical secondary structure and the increased random coil content as a result of the Maillard reaction, as reported in section 3.4. The Maillard reaction also enhanced the surface hydrophilicity of the conjugates, thus improving their solubility and stronger affinity for water. Qu et al. (2018) reported on the steric stability effect of cocoyam mucilage, which might have contributed to the higher solubility of the conjugated protein when compared to the protein isolate. These data follow a similar trend to that of Ma et al. (2019). This study suggested that *Lagenaria siceraria* protein isolate‐cocoyam mucilage Maillard conjugates might be a potential ingredient in food emulsions.

### 3.8 *Lagenaria siceraria* Protein Isolate and Its Conjugates' Emulsifying Qualities

**FIGURE 5:**
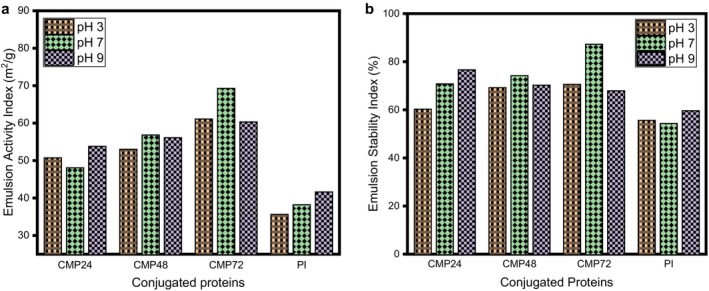
Emulsifying properties of *Lagenaria siceraria* protein isolate‐cocoyam mucilage conjugate (a) emulsion activity index (b) emulsion stability index. *N* = 3; values are mean ± SD. Bars with different alphabets are statistically different (*p* < 0.05) at the studied pHs. CMP24: *Lagenaria siceraria* seed protein isolate‐cocoyam mucilage conjugate at 24 h. CMP48: *Lagenaria siceraria* seed protein isolate‐cocoyam mucilage conjugate at 48 h. CMP72: *Lagenaria siceraria* seed protein isolate‐cocoyam mucilage conjugate at 72 h. PI = *Lagenaria siceraria* seed protein isolate.

The emulsifying activity index (EAI) of modified *Lagenaria siceraria* protein isolate was significantly (*p* ≤ 0.05) higher than that of its unmodified counterpart across all pH values; as the glycation period increased (Figure 5a). As the conjugation reaction progressed with glycation time, the emulsion stability index increased at all pH levels, particularly at pH 7 (Figure 5b). An increase in EAI in the conjugated samples suggests that the ideal glycation time for obtaining superior emulsion stability was at 72 h. The conjugates had higher emulsifying stability and activity indices, demonstrating that the Maillard reaction enhances the emulsifying properties of proteins. According to Shen and Li 2021, the modified protein isolate exhibited greater emulsion droplet steric stability than untreated proteins. Therefore, conjugating *Lagenaria siceraria* protein isolate with cocoyam mucilage enhances its potential use in the food system, such as the dairy industry, for ice cream and salad dressings.

### 3.9 Foaming Characteristics of *Lagenaria siceraria* Protein Isolate and Its Conjugates

**FIGURE 6:**
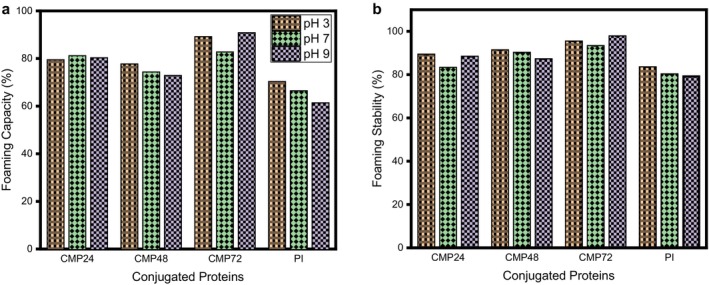
Foaming characteristics of *Lagenaria siceraria* protein isolate‐cocoyam mucilage conjugate (a) Foaming capacity (b) Foaming stability. *N* = 3; values are mean ± SD. Bars with different alphabets are statistically different (*p* < 0.05) at the studied pHs. CMP24: *Lagenaria siceraria* seed protein isolate‐cocoyam mucilage conjugate at 24 h. CMP48: *Lagenaria siceraria* seed protein isolate‐cocoyam mucilage conjugate at 48 h. CMP72: *Lagenaria siceraria* seed protein isolate‐cocoyam mucilage conjugate at 72 h. PI = *Lagenaria siceraria* seed protein isolate.

An overall increase in foaming capacity with conjugation, especially at pH 9, and a minor improvement in foaming stability were observed (Figure 6a,b). Compared to the unmodified *Lagenaria siceraria* protein isolate (PI), the conjugates showed a significantly higher foaming capacity (*p* ≤ 0.05). This higher foaming capacity may be attributed to improved solubility, which allows faster absorption during the whipping process and produces stronger foam. This contrasts with the report by Shen and Li (2021), which found that acylation of pea proteins reduced foaming capacity and stability compared with unmodified proteins. However, the results of this study align with the findings of Liu et al. (2012), who reported improved foaming properties of peanut protein conjugates. Additionally, improved solubility may account for the enhanced foaming capacity, particularly at pH 9, as it may be absorbed more quickly during whipping to produce more foam than samples with lower solubility (Zhao et al. 2013). The modified *Lagenaria siceraria* proteins did not differ significantly in foaming stability (FS), except for the *Lagenaria siceraria* protein isolate‐cocoyam mucilage glycated for 48 and 72 h at pH 7 and 9. This may be due to an increase in net charge density, which may decrease interactions between proteins in the foam and prevent the formation of an elastic film at the air‐water interface (Arogundade and Adebowale 2013). It may also be due to excessive Maillard progression, aggregation effects, and structural collapse at longer reaction times. The pH‐dependent behavior, which can be linked to changes in protein structure and the degree of interaction between the protein and the polysaccharides at different pH levels, suggests that alkaline environments favor the formation of stable foams. These insights facilitate the customization of protein‐based ingredients for specific food applications, including drinks and desserts, where foamy properties are needed.

## 4 Conclusion

The research demonstrated that glycation with cocoyam mucilage significantly improves the functional characteristics of the *Lagenaria siceraria* protein isolate through the Maillard reaction. Methionine, a limiting amino acid in most legumes, was present at higher concentrations in the *Lagenaria siceraria* protein isolate. Both essential and non‐essential amino acid values were comparable to FAO/WHO‐recommended values, making the isolate suitable for various food formulations, including low‐calorie, high‐fiber foods, emulsions, and foamy desserts. This work also demonstrated that β‐sheets and α‐helices dominate the secondary structure of the protein isolates and their conjugates. Conjugation and reaction duration increased the α‐helical content while decreasing the β‐sheet content in the modified samples relative to the protein isolate. SEM analysis of the conjugates' microstructure revealed that time and glycation affected the surface structure and validated the protein‐polysaccharide interaction. These findings suggest that the cocoyam mucilage Maillard conjugate and the protein isolate from *Lagenaria siceraria* represent a viable alternative for plant‐based, sustainable protein components in functional food sectors. The inability to confirm the direct molecular weight of the conjugates is a limitation of the research. The study relies on indirect indicators of glycation, such as a decrease in lysine, an increase in Amadori compounds, FTIR changes, and functional improvements. While these indicators suggest glycation, they do not confirm covalent conjugate formation.

## Author Contributions

**Gbenga Isaac Oluwafemi:** investigation, writing – original draft, methodology, formal analysis. **Eunice Moriyike Ogunbusola:** conceptualization, supervision, writing – review and editing, methodology. **Kudirat Titilope Araoye:** formal analysis, methodology. **Opeyemi Olaitan Alabi:** conceptualization, writing – review and editing, formal analysis, investigation. **Kunle Oni:** supervision, validation.

## Funding

The authors have nothing to report.

## Conflicts of Interest

The authors declare no conflicts of interest.

## Data Availability

The data that support the findings of this study are available from the corresponding author and co‐authors upon reasonable request.
